# Gender-Specific Mechanisms Underlying the Amelioration of High-Fat Diet-Induced Glucose Intolerance in B-Cell-Activating Factor Deficient Mice

**DOI:** 10.1371/journal.pone.0166225

**Published:** 2016-11-04

**Authors:** Bobae Kim, Chang-Kee Hyun

**Affiliations:** School of Life Science, Handong Global University, Pohang, Gyungbuk, Republic of Korea; Brown University Warren Alpert Medical School, UNITED STATES

## Abstract

It has recently been found that B cell activating factor (BAFF) plays an important role in the regulation of energy homeostasis. We also have previously reported that BAFF deficiency reverses high-fat (HF) diet-induced glucose intolerance by potentiating adipose tissue function. In the present study, we found that BAFF deficient (BAFF^-/-^) mice exhibit gender-specific differences in protection against diet-induced glucose intolerance, and aimed to characterize the gender-dependent molecular alterations in energy metabolism. Under HF feeding conditions, serum BAFF level of female wild-type (WT) mice was considerably higher than that of male mice. Despite increased body weight gain, both male and female BAFF^-/-^ mice showed significantly improved glucose tolerance compared to their WT counterparts. Expressions of genes involved in glucose transport, thermogenesis and lipid oxidation were up-regulated in brown adipose tissues of both male and female BAFF^-/-^ mice. Interestingly, the expression of thermogenic genes in subcutaneous adipose tissue was significantly enhanced in female BAFF^-/-^ compared to WT mice, but the difference was not observed between male BAFF^-/-^ and WT mice. The enhanced thermogenic program was confirmed by higher protein levels of UCP1 and irisin in female BAFF^-/-^ than in WT mice. Additionally, adiponectin production in white adipose tissues and AMPK phosphorylation in subcutaneous adipose tissue were also significantly elevated in female BAFF^-/-^ compared to WT mice, but not in male BAFF^-/-^ mice. Our findings define a comprehensive scenario for the enhancing effect of BAFF depletion on glucose tolerance wherein the underlying mechanism is, at least in part, gender-specific, and suggest that gender difference should be considered as an important factor in the use of BAFF blockade as a therapeutic approach for the prevention and treatment of type 2 diabetes.

## Introduction

B-cell-activating factor (BAFF) is a tumor necrosis factor ligand family protein which promotes B-cell survival and development, which is also a ligand for receptors including BAFF receptor (BAFF-R), B-cell maturation antigen (BCMA), and transmembrane activator and CAML interactor (TACI) [1]. Being synthesized in a form of membrane-bound protein and released as a soluble cytokine by proteolytic cleavage, BAFF binds to its receptors to activate classical or alternative NF-κB pathway and regulate the expression of genes involved in B-cell differentiation and proliferation [2]. Several studies have demonstrated that both BAFF and BAFF receptors are expressed in a variety of cell types, including mature adipocytes, playing a role in regulation of energy homeostasis, and proposed that the blockade of BAFF can be considered as a therapeutic approach for the treatment of metabolic diseases [3–5]. Previously, we reported that insulin resistance in BAFF knockout (BAFF^-/-^) male mice was significantly improved in spite of diet-induced weight gain, which was found to be due to up-regulated metabolic functions of brown and white adipose tissues mediated by FGF21 and leptin [6].

It has also been reported that BAFF and its receptors act as trophic factors in lymphocyte malignancies and immune-related disorders, such as systemic lupus erythematosus (SLE), Sjögren’s syndrome, and rheumatoid arthritis (RA), which are characterized by the production of pathogenic autoantibodies against certain nuclear antigens and DNA [7–9]. Interestingly, these autoimmune diseases exhibit a strong sex bias in patients and mouse models in common: SLE develops at a female-to-male ratio of 9:1, Sjögren’s syndrome at a ratio of 9–15:1, and RA at a ratio of 3:1 [10,11]. The mechanism underlying this gender-specific pathogenesis of autoimmunity is likely to involve immunomodulatory actions of sex hormones as well as non-hormonal factors encoded by genes on the X or Y chromosomes [12,13]. Indeed, it has been shown that receptors for estrogens regulate cell development and signaling pathways of the innate and adaptive immune system [14]. Likewise, murine BAFF expression is also found to be up-regulated by estrogen and interferons through p202 protein, by which the contribution of BAFF expression to a sex bias in the development of autoimmunity was demonstrated [15]. Based on these findings of recent studies, we hypothesized that BAFF expression could be associated with a gender-specific insulin sensitization in BAFF depleted mice.

In this study, we examined the impact of BAFF depletion on the development of glucose intolerance using BAFF^-/-^ male and female mice fed a high-fat (HF) diet. Our results confirmed that glucose tolerance was significantly improved by BAFF depletion in both sexes, which was attributed by the enhancement of lipid metabolism in brown adipose tissue. Contrary to the male mice, however, female BAFF^-/-^ mice showed enhanced thermogenic capacity of subcutaneous adipose tissue compared to female wild-type (WT) controls, which was associated with an increase in the level of irisin production in skeletal muscle. BAFF depletion also promoted adipose tissue adiponectin production and serum adiponectin concentration in female mice, leading to enhanced AMP-activated protein kinase (AMPK) phosphorylation in subcutaneous adipose tissue. Our study suggests that sex difference should be considered as a critical factor to describe the underlying mechanism responsible for the improvement in glucose tolerance due to BAFF depletion, and gender-specific therapeutic application of BAFF inhibition could be a novel strategy for the treatment of type 2 diabetes.

## Materials and Methods

### Animals

Male and female C57BL/6J wild-type (BAFF^+/+^) and BAFF-deficient (BAFF^-/-^) mice were purchased from Central Laboratory Animal Inc. (Seoul, Korea), and The Jackson Laboratory (Bar Harbor, ME; stock number 010572), respectively. Systemic BAFF knockout was executed and confirmed as described previously [6]. Mice were maintained under a 12 h light:dark cycle at a constant temperature of 22 ± 1°C and humidity of 45 ± 10%. To stabilize all metabolic conditions, 5-week-old male and female mice were fed normal chow diet (2018S, Harlan Laboratories, Indianapolis, IN) and individually housed in cages for a week. After the stabilization, mice were switched to the HF diet containing 60%kcal from fat (D12493, Research Diets Inc., NJ) for 5 weeks.

Mice were fasted for 4 h and sacrificed by cervical dislocation. Tissues of the liver, spleen, subcutaneous adipose tissue, gonadal adipose tissue, and interscapular brown adipose tissue were harvested, snap-frozen in liquid nitrogen, and stored at -70°C until processed for RNA and protein analysis. All the experimental protocols were approved by the Committee on the Ethics of Animal Experiments of the Handong Global University (permit number: 20151022–010).

### Glucose tolerance test

After 4 weeks of HF feeding, mice were fasted for 16 h and followed by intraperitoneal injection of glucose (2 g/kg). Blood samples were obtained by tail-bleeding, and glucose levels were measured at 0, 15, 30, 60, 90 and 120 min after glucose injection by Accu-Check Go (Roche Diagnostics GmbH, Basel, Switzerland).

### Western blotting

Western blotting was performed as described previously [16,17]. Serum sample was diluted 10-fold with reducing protein sample buffer, heated at 95°C for 10 min and 10μl of the sample was analyzed by SDS-PAGE-immunoblotting assay. Antibodies against adiponectin, phospho-AMPK (Thr172), total AMPK, (Cell signaling technology, Beverly, MA) GAPDH (Bioss antibodies, Woburn, MA), irisin (Aviscera Bioscience, Santa Clara, CA), phospho-PPARγ (Ser112) and UCP1 (Abcam, Cambridge, UK) were used as primary antibodies, followed by the appropriate IgG-HRP conjugated secondary antibody. Proteins were visualized by ECL.

### Real-time RT PCR

Total RNA was extracted using TRI reagent (Molecular Research Center, Cincinnati, OH) and reverse transcribed with oligo (dT) primer and GoScript^TM^ reverse transcription system (Promega, Madison, WI). Quantitative PCR of gene transcripts for Acyl-CoA oxidase 1 (Acox1), adiponectin, BAFF, carnitine palmitoyltransferase 1 (CPT1), fibroblast growth factor 21 (FGF21), glucose transporter 1 (GLUT1), GLUT4, leptin, mitochondrially encoded NADH dehydrogenase 5 (ND5), peroxisome proliferator-activated receptor γ coactivator 1α (PGC1α), peroxisome proliferator-activated receptor α (PPARα), PR domain containing 16 (Prdm16) and uncoupling protein 1 (UCP1) was performed by using gene-specific primers. Primer sequences are available upon request. Results were presented as mean ± S.D. normalized to expression of 36B4 (Arbp) using the ΔΔCt method.

### Statistics

All data were presented as mean ± S.D. Comparisons of two groups were performed by two-tailed Student’s t-test. *p* values < 0.05 were considered as statistically significant.

## Results

### BAFF production level is higher in female compared to male mice under HF diet condition

Comparison of serum BAFF level between male and female WT mice on HF diet revealed a significant increase of BAFF production in female HF diet-fed mice relative to male counterparts (S1A Fig). Since BAFF is known to be expressed by immune cells as well as adipocytes, BAFF mRNA expression level was measured in various tissues including the spleen, liver, skeletal muscle, and several adipose tissues. We observed that the expression in skeletal muscle and the liver was negligible compared to that in the spleen or adipose tissues. Interestingly, gonadal adipose tissue of female mice showed significantly higher level of BAFF mRNA expression than that of male mice, whereas splenic expression was substantially decreased in female compared to male mice (S1B Fig). BAFF protein level in gonadal adipose tissue was also significantly higher in female than male mice, which was consistent with the mRNA expression result (S1C Fig). The levels of BAFF mRNA in subcutaneous adipose tissue and interscapular brown adipose tissue were not different between female and male.

### BAFF deficiency improves glucose tolerance under HF feeding condition despite increased adiposity

**Fig 1:**
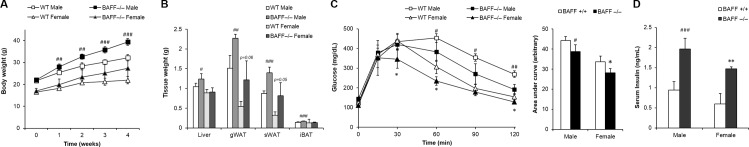
BAFF^-/-^ mice display enhanced glucose tolerance under HF diet-fed conditions despite increased adiposity. BAFF^-/-^ and wild-type C57BL/6J mice were fed a high-fat diet for 5 weeks. (A) Changes of body weight for 4 weeks of HFD feeding (n = 7~8). (B) Changes of tissue weight after 5-week HFD feeding (n = 7~8). (C) Glucose tolerances in after 4 weeks on HF diet (n = 6~7) and the area under the curve of GTT. The blood glucose levels were measured at 0, 15, 30, 60, 90 and 120 after intraperitoneal injection of glucose (2 g/kg). (D) Serum concentration of insulin quantified by ELISA. Serum sample was analyzed according to the manufacturer’s protocol. Data represent means ± SD. *p < 0.05 between female WT and female BAFF^-/-^ mice. #*p* < 0.05, ##*p* < 0.01, and ###*p* < 0.001 between male WT and male BAFF^-/-^ mice. gWAT: gonadal white adipose tissue, sWAT: subcutaneous white adipose tissue, iBAT: interscapular brown adipose tissue.

After 5 weeks of HF feeding, both male and female BAFF^-/-^ mice showed increased body weight gain compared to their WT controls, which was particularly prominent in male mice (Fig 1A). There was no difference in food intake between WT and BAFF^-/-^ mice of both sexes, which indicates that the higher body mass gain in BAFF^-/-^ mice compared to WT controls was not due to a higher energy intake (S2A Fig). Analysis of tissue weight changes revealed that expansion of adipose tissues mainly contributed to the increase in weight gain (Fig 1B). Interestingly, weights of the liver and interscapular brown adipose tissue were significantly higher in male BAFF^-/-^ mice relative to male WT mice, while this difference was not observed between female BAFF^-/-^ and WT counterparts. Consistent with our previous study [6], BAFF depletion enhanced glucose tolerance (Fig 1C) with elevated serum insulin level (Fig 1D). Despite HF diet-induced adiposity, both male and female BAFF^-/-^ mice showed significantly improved glucose tolerance compared to WT controls.

### BAFF deficiency alters the expression of genes involved in glucose transport and thermogenic program in brown adipose tissue

**Fig 2:**
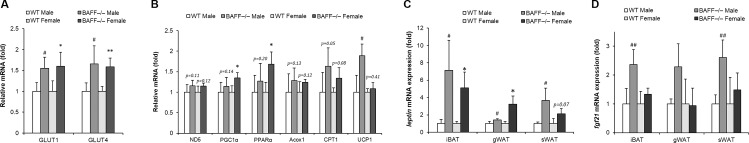
BAFF deficiency enhances expression of genes involved in glucose transport and thermogenic program in brown adipose tissue. (A) Effect of BAFF deficiency on glucose transporter gene expression in brown adipose tissue. (B) Effect of BAFF deficiency on expression of thermogenic and lipid oxidative genes in brown adipose tissue. (C and D) Effect of BAFF deficiency on leptin and FGF21 mRNA expression level in adipose tissues. Total RNA was isolated from brown, gonadal and inguinal adipose tissues of mice with HF feeding and mRNA expression level was analyzed. Gene expression level is normalized with mRNA expression level of Arbp (n = 4~5). *p < 0.05 and **p < 0.01 between female WT and female BAFF^-/-^ mice. #*p* < 0.05 and ##*p* < 0.01 between male WT and male BAFF^-/-^ mice. iBAT: interscapular brown adipose tissue, gWAT: gonadal white adipose tissue, sWAT: subcutaneous white adipose tissue.

In the previous study, we reported that BAFF depletion enhanced the function of brown adipose tissue in HF diet-fed male mice, which was found to be mainly mediated by FGF21 and leptin [6]. Analysis of the gene expression in brown adipose tissues revealed significant increases in the expression of glucose transporters, GLUT1 and GLUT4 (Fig 2A), as well as modestly increased mRNA expressions in thermogenic regulators and lipid oxidative enzymes, including ND5, PGC1α, PPARα, Acox1 and CPT1, in both male and female BAFF^-/-^ mice compared to their WT counterparts (Fig 2B). The mRNA level of UCP1 was significantly elevated in male BAFF^-/-^ mice, but this elevation was not observed in female BAFF^-/-^ mice. Leptin levels are proportional to fat mass, and we observed increased leptin levels in adipose tissues in both male and female BAFF^-/-^ mice (Fig 2C) exhibiting increased fat mass, indicating that there is no sex difference in leptin expression. However, although the up-regulation of FGF21 mRNA expression was observed in male BAFF^-/-^ mice relative to WT controls, there was, unexpectedly, no difference between female BAFF^-/-^ and WT mice (Fig 2D). This observation was not made only in brown adipose tissue, but also in gonadal and subcutaneous white adipose tissues.

### BAFF deficiency alters the expression of genes involved in glucose metabolism and thermogenic program in subcutaneous adipose tissue of female mice

**Fig 3:**
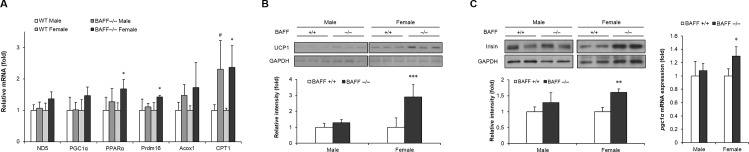
Female BAFF^-/-^ mice show enhanced thermogenic program in subcutaneous adipose tissue. (A) Effect of BAFF deficiency on mRNA expression of thermogenic and lipid oxidative genes in subcutaneous adipose tissue. Total RNA was isolated from inguinal adipose tissue of mice with HF feeding and mRNA expression levels were analyzed. All genes are normalized with mRNA expression level of Arbp (n = 4~5). (B) Effect of BAFF deficiency on UCP1 protein level in subcutaneous adipose tissue. (C) Effect of BAFF deficiency on irisin protein level and PGC1α mRNA level in quadriceps muscle. Proteins were extracted from the tissues for SDS-PAGE-immunoblot analysis. *p < 0.05, **p < 0.01 and ***p < 0.001 between female WT and female BAFF^-/-^ mice. #*p* < 0.05 between male WT and male BAFF^-/-^ mice.

Beige adipocytes are defined by their multilocular lipid droplet morphology, high mitochondrial content and the expression of a core set of brown fat-specific genes [18]. Accumulation of beige adipocytes in white adipose tissue, which is referred to as ‘browning’, could be triggered by various stimuli [19], and it contributes to heat production and attenuation of metabolic disease [20]. To assess the effect of BAFF depletion on thermogenic capacity of subcutaneous adipose tissue, we measured mRNA expression of genes involved in lipid metabolism, mitochondrial function, and thermogenic program. Under HF feeding condition, both male and female BAFF^-/-^ mice showed increased PPARγ activity by preventing its phosphorylation at Ser112, as well as up-regulated expressions of lipogenic and adipogenic genes, such as CD36, C/EBPα, PPARγ, TLE3 and SCD1, compared to their WT counterparts (S3A Fig and S3B Fig) in subcutaneous adipose tissue, showing no male-female difference. On the contrary, female, but not male, BAFF^-/-^ mice had significantly higher mRNA levels of genes involved in mitochondrial function and thermogenesis including PPARα and Prdm16, and moderately higher levels of ND5 and PGC1α than WT controls (Fig 3A). Unlike subcutaneous adipose tissue, this conspicuous alteration of mRNA expression was not observed in gonadal white adipose tissue (S3C Fig). Commensurate with the increased mRNA expression of thermogenic genes in subcutaneous adipose tissue, female, but not male, BAFF^-/-^ mice had substantially higher level of UCP1 than WT controls (Fig 3B). To confirm that BAFF depletion enhanced thermogenic program in subcutaneous adipose tissue of female mice, but not in males, we have additionally performed a cold-induced thermogenesis experiment. As a result, we have found that expression of thermogenic program genes such as UCP1, ND5 and PGC1α in subcutaneous adipose tissue of female mice, but not male mice, fed a normal chow diet was substantially increased in response to cold stimulation for 96 h (S4 Fig). Particularly, the cold-induced UCP1 expression in female BAFF^-/-^ mice was significantly higher than that in their WT counterparts whereas there was no increase in UCP1 expression in cold-exposed male BAFF^-/-^ mice compared to their WT controls. These observations suggest that the enhancement of thermogenic capacity in subcutaneous adipose tissue by BAFF depletion was significant in female mice, but not in males.

To further understand how BAFF deficiency promotes thermogenesis in subcutaneous white adipose tissue of female mice, we next analyzed protein level of irisin, which is an exercise-inducible myokine acting as an endocrine activator for adipose tissue browning [21]. We observed that the level of irisin was remarkably increased in skeletal muscle of female BAFF^-/-^ mice compared to WT controls, which was accompanied by a substantial increase of PGC1α mRNA level (Fig 3C). Again however, contrary to female mice, this effect was not observed in male BAFF^-/-^ mice.

### Female BAFF^-/-^ mice display enhanced adiponectin production and AMPK phosphorylation in subcutaneous adipose tissue

**Fig 4:**
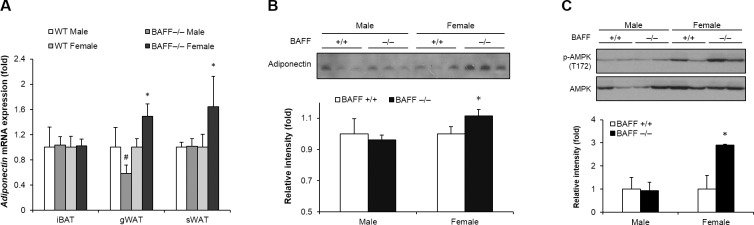
Female BAFF^-/-^ mice show enhanced adiponectin production and AMPK phosphorylation in subcutaneous adipose tissue. (A) Effect of BAFF deficiency on adiponectin mRNA expression level in adipose tissues. Total RNA was isolated from interscapular brown, gonadal and subcutaneous adipose tissues of mice with HF feeding and adiponectin mRNA expression level was analyzed. All genes are normalized with mRNA expression level of Arbp. (B) Effect of BAFF deficiency on serum adiponectin protein level. (C) Effect of BAFF deficiency on AMPK phosphorylation in subcutaneous adipose tissue. Serum sample diluted 10-fold with protein sample buffer or proteins extracted from subcutaneous were analyzed by SDS-PAGE-immunoblot assay. Data represent means ± SD (n = 4~5). *p < 0.05 between female WT and female BAFF^-/-^ mice. #*p* < 0.05 between male WT and male BAFF^-/-^ mice. gWAT: gonadal white adipose tissue, sWAT: subcutaneous white adipose tissue, iBAT: interscapular brown adipose tissue.

Adiponectin is an adipokine which stimulates fatty acid oxidation and enhances glucose tolerance of peripheral tissues [22]. We observed that adiponectin gene expression was up-regulated in white adipose tissues, but not in brown adipose tissue, of female BAFF^-/-^ mice compared to WT controls (Fig 4A). Consistent with the data on mRNA expression, serum adiponectin concentration was significantly increased in female BAFF^-/-^ relative to WT controls (Fig 4B), which was not detected in male mice. As adiponectin activates AMP-activated protein kinase (AMPK) by promoting its phosphorylation leading to increased glucose uptake, fatty acid oxidation and thermogenic program, we next measured the level of AMPK phosphorylation in adipose tissues and skeletal muscle. Notably, phosphorylated AMPK level was significantly elevated in subcutaneous adipose tissues of female BAFF^-/-^, but not of male mice, relative to WT mice (Fig 4C). However, in gonadal and brown adipose tissues and skeletal muscle, AMPK phosphorylation remained unchanged.

## Discussion

Recent studies have reported that BAFF and its receptors are not only immunotropic factors but also regulatory factors of energy metabolism [3–5]. Interestingly, BAFF-related autoimmune diseases, described by the pathogenic production of autoantibodies, are known to have a strong sex bias in both patients and mouse models [10,11]. Indeed, development of adiposity and metabolic syndrome shows gender-specific differences attributed by sex hormones [23–25]. The goal of this study was to elucidate the gender-specific mechanism of BAFF deficiency in metabolic regulation and glucose tolerance using male and female BAFF^-/-^ mice fed HF diet.

It has been reported that plasma BAFF levels are gender-dependent, proportional to BMI, percentage of body fat, and significantly different between obese and non-obese individuals [26]. The obese females presented higher BAFF levels compared with non-obese females or obese males. In this study, we observed that female WT mice, after 5 weeks of HF feeding, had significantly higher serum BAFF concentration than male WT mice (S1A Fig). Patterns of BAFF mRNA expression were different between tissue types, with enriched expression in the spleen and adipose tissues, yet near absence in skeletal muscle and the liver, indicating that the spleen and adipose tissues are major resources of serum BAFF (S1B Fig). We also found that female mice on a HF diet had significantly higher level of BAFF in gonadal white adipose tissue than male mice, whereas splenic BAFF mRNA level was substantially lower in female than male mice (S1B Fig and S1C Fig). This suggests that, in female mice, the consumption of HF diet resulted in an increased level of serum BAFF, which was due to enhanced BAFF production in gonadal adipose tissue, not in the spleen. Considering the higher serum BAFF level in female mice, we hypothesized that the phenotypes elicited by the HF diet in BAFF-deficient female mice might be more remarkable than those observed in male counterparts.

Under HF dietary conditions, both male and female BAFF^-/-^ mice showed significantly improved glucose tolerance compared to their WT control mice (Fig 1C), in spite of increased body weights (Fig 1A). However, insulin tolerance test did not show significant difference between WT and BAFF^-/-^ mice (S2B Fig), suggesting that BAFF deletion-induced improvement of glucose tolerance was due to enhanced serum insulin level, not insulin sensitivity. Indeed, serum insulin level was significantly elevated in both male and female BAFF^-/-^ mice (Fig 1D). Consistent with our previous report, weights of the liver, white adipose tissues and brown adipose tissue in male BAFF^-/-^ mice were significantly higher compared to male WT controls (Fig 1B). In contrast, the extent of body and tissue weight gain in female BAFF^-/-^ compared with WT control mice was less significant than that in male mice. These different responses to HF feeding between male and female BAFF^-/-^ mice can be explained by the anti-obesity action of estrogen and gender-specific effects of sex hormones on adipose tissue distribution [27,28].

Our previous report demonstrated that BAFF deficiency prevents mice from HF diet-induced glucose intolerance at least in part by potentiating brown adipose tissue function, particularly through enhancement of FGF21 expression and leptin action [6]. In this study, it was additionally found that gene expression of glucose transporters in brown adipose tissue, GLUT1 and GLUT4, was significantly elevated in both male and female BAFF^-/-^ mice compared to their WT controls (Fig 2A), which also could account for the improvement of glucose tolerance.

Activation of brown adipose tissue, a thermogenic organ which dissipates heat using lipid as an energy source, confers beneficial effects on adiposity, glucose intolerance and hyperlipidaemia [20]. We addressed this issue in this study and evaluated the beneficial effect of BAFF deficiency on thermogenic program under HF dietary condition. Consistent with the conclusion of our previous study, we observed modestly increased mRNA expressions of genes for mitochondrial ND5, thermogenic regulators such as PGC1α and PPARα, and lipid oxidative enzymes such as Acox1 and CPT1 in brown adipose tissue of BAFF^-/-^ mice compared to WT controls (Fig 2B). Notably, UCP1 expression was significantly upregulated in male BAFF^-/-^ mice, but not in female BAFF^-/-^ mice. These results suggest that mitochondrial biogenesis and thermogenic capacity are augmented by BAFF depletion, however, the augmenting effect of UCP1 expression is exerted only in male mice, indicating that activation of BAT function is male-specific mechanism. FGF21 is a peptide hormone which induces thermogenic response in brown adipose tissue to adrenergic stimulation [29]. In our previous study, it was found that male BAFF^-/-^ mice had a higher FGF21 expression in adipose tissues than WT controls [6]. However, in contrast to the case of male mice, no significant difference in FGF21 expression was observed between female BAFF^-/-^ and WT mice (Fig 2D), suggesting that FGF21-mediated activation of brown adipose tissue is a male-specific mechanism underlying insulin-sensitizing effect of BAFF depletion, but not for female mice.

Similar to adipocytes in brown adipose tissue, an increase of UCP1-expressing beige adipocytes with thermogenic capacity in white adipose tissue also exerts beneficial effects against metabolic diseases [20]. Beige adipocytes are clustered and located primarily in murine subcutaneous adipose tissue, and their development is regulated by various factors including Prdm16 and PGC1α [18]. In this study, we found that the mRNA expression of genes involved in mitochondrial function and thermogenesis, including ND5, PGC1α, PPARα and Prdm16, was significantly elevated in subcutaneous adipose tissue of female BAFF^-/-^ mice compared to WT controls, but the difference was not observed in male mice (Fig 3A). This female-specific tendency was corroborated by the observation of an elevated UCP1 protein level in subcutaneous adipose tissue of female BAFF^-/-^ mice, but not of male mice (Fig 3B). The female-specific elevation of thermogenesis in subcutaneous adipose tissue was also confirmed by a cold-induced thermogenesis experiment, which showed a significantly increased UCP1 expression in response to cold exposure in subcutaneous adipose tissue of female, but not male, BAFF^-/-^ mice compared to their WT counterparts (S4 Fig). These findings suggest that the protective effect of BAFF deficiency on glucose intolerance in female mice is, at least in part, elicited via improved thermogenic capacity in subcutaneous adipose tissue. This conclusion appears to be inconsistent with the observed higher adipose mass of subcutaneous adipose tissue in BAFF^-/-^ mice than WT controls. However, this conflict can be explained by the enhancement of lipogenic activity in adipose tissues, which might mask the impact of elevated energy expenditure and result in increased lipid accumulation. Our data show that, although the browning effect of BAFF depletion in subcutaneous adipose tissue was evident (Fig 3), the lipogenic and adipogenic activity was also enhanced in subcutaneous adipose tissue of BAFF^-/-^ mice (S3A Fig and S3B Fig). We observed not only significantly higher expressions of lipogenic genes (S3A Fig) but also a significantly reduced phosphorylation of PPARγ at serine 112 in subcutaneous adipose tissue of BAFF^-/-^ mice compared to WT controls (S3B Fig). PPARγ phosphorylation at serine 112 is known to suppress its activity by modulating its ligand binding affinity and cofactor recruitment, and mice homozygous for the S112A mutant were protected from diet-induced adiposity [30]. Therefore, our observations provide an explanation for the increase of adipose mass in subcutaneous adipose tissue of BAFF^-/-^ mice despite enhanced thermogenic capacity.

Irisin, the cleaved and secreted portion of fibronectin domain-containing protein 5 (FNDC5), is a soluble peptide hormone expressed mainly in skeletal muscle and stimulates browning of white adipocytes, of which expression is regulated by PGC1α [31,32]. Moreno-Navarrete et al. reported that, in obese patients, FNDC5 gene expression in muscle was significantly decreased in association with type 2 diabetes, and interestingly, muscle FNDC5 expression was significantly associated with UCP1 expression in adipose tissue [33]. In this study, we observed that protein level of irisin in quadriceps muscle of female, but not male, BAFF^-/-^ mice was substantially higher than that of WT controls, which was accompanied with significantly increased PGC1α mRNA level in quadriceps muscle (Fig 3C). These data suggest that, in female mice, the enhancing effect of BAFF deficiency on glucose tolerance is possibly mediated by irisin-induced activation of thermogenic program in subcutaneous adipose tissue. It has been reported that circulating irisin level is positively correlated with serum estradiol level [34], and estrogen receptor related receptor α (ERRα) interacts with PGC1α-regulating gene transcriptions related to energy metabolism [35]. These previous studies provide a plausible mechanism for the female-specific enhancement of irisin production in BAFF deficient mice. However, how BAFF deficiency mediates the enhanced irisin production in female mice is still unclear and the underlying mechanism remains to be elucidated.

Adiponectin is a metabolically favorable adipokine that improves glucose tolerance by increasing energy expenditure and fatty acid oxidation through activation of AMPK [22]. Recently, it has been reported that adiponectin enhances cold-induced browning and thermogenic program of subcutaneous adipose tissue [36]. Furthermore, it has been shown that the genes transcriptionally regulated by AMPK and those by PGC1α are largely overlapping each other, suggesting that PGC1α acts as an important mediator of AMPK-induced gene expression [37]. Several studies have provided evidence that AMPK activation leads to an increase in PGC1α expression in skeletal muscle and adipose tissue, and AMPK requires PGC1α activity to modulate the expression of several key players in mitochondrial and glucose metabolism [38,39]. In the present study, levels of adiponectin mRNA expression in white adipose tissues (Fig 4A) and serum adiponectin were significantly higher in female, but not male, BAFF^-/-^ mice than that of WT controls (Fig 4B). These observations were corroborated in subcutaneous adipose tissue which showed significantly enhanced AMPK phosphorylation in female BAFF^-/-^ mice compared to WT controls (Fig 4C). Taken together, these data suggest that up-regulation of adiponectin increases AMPK activation, leading to enhanced expression of PGC1α and other thermogenic genes in subcutaneous adipose tissue in HF diet-fed BAFF-deficient female mice. More studies elucidating the relationship between BAFF, sex hormones and adipokines are needed to fully understand this gender-specific effects of BAFF depletion.

In summary, our findings in the current study reveal that the protective effect of BAFF deficiency from HF diet-induced glucose intolerance is partly gender-specific. Although both male and female BAFF-deficient mice had improved glucose tolerance despite of increased adiposity, which was associated with enhanced function of brown adipose tissue, the FGF21-mediated effect was shown only in male mice, not in females. Instead, female BAFF-deficient mice displayed an increase in thermogenic gene program and browning of subcutaneous adipose tissue induced by irisin, but not in male mice. In addition, BAFF depletion in female, but not male, mice resulted in the enhancement of adiponectin production in white adipose tissues, leading to increased AMPK phosphorylation and expression of thermogenic and lipid oxidative genes in subcutaneous adipose tissue. Our findings suggest that gender-specific dimorphic pattern should be considered as a critical factor to understand the mode of action underlying the protective effect of BAFF depletion against HF diet-induced glucose intolerance. These results, together with those obtained in our previous study [6], also have positive implications for the application of BAFF blockade to the treatment of glucose intolerance.

## Supporting Information

S1 Fig Female WT mice have elevated levels of BAFF production under HF diet condition compared to male counterparts. (A) Serum BAFF concentration quantified by ELISA. Serum sample were diluted 10–fold with dilution buffer, and analyzed according to the manufacturer’s protocol. (B) Levels of BAFF mRNA expression in various tissues of male and female mice. Total RNA was isolated from the spleen, liver, skeletal muscle, gonadal, inguinal, and brown adipose tissues of mice with HF feeding and BAFF mRNA expression levels were analyzed. (C) BAFF concentration in gWAT quantified by ELISA. Tissue lysates were diluted 2-fold for ELISA analysis. Total RNA was isolated from the liver, spleen, quadriceps, gonadal, inguinal, and brown adipose tissues of mice with HF feeding and BAFF mRNA expression levels were analyzed. The mRNA expression level of BAFF is normalized with mRNA expression level of Arbp. Data represent means ± SD (n = 4~5). **p < 0.01 between female WT and female BAFF^-/-^ mice. gWAT: gonadal white adipose tissue, sWAT: subcutaneous white adipose tissue, iBAT: interscapular brown adipose tissue.
(TIF)

S2 Fig There are no differences in calorie intake and insulin sensitivity between WT and BAFF^-/-^ mice. BAFF^-/-^ and WT mice were fed a high-fat diet for 5 weeks. (A) Average daily calorie intake for 4 weeks of HFD feeding (n = 7~8). (B) Insulin tolerance of male WT and BAFF^-/-^ mice after 4 weeks on HF diet (n = 5). The blood glucose levels were measured at 0, 15, 30, 60, 90 and 120 after intraperitoneal injection of insulin (0.75U/kg). Data represent means ± SD.
(TIF)

S3 Fig Female WT mice shows altered gene expressions of lipid metabolism in adipose tissues compared to male counterparts under HF diet condition. (A) Effect of BAFF deficiency on mRNA expression of lipogenic and adipogenic genes in subcutaneous white adipose tissue. (B) Effect of BAFF deficiency on PPARγ phosphorylation in subcutaneous white adipose tissue. Proteins extracted from inguinal adipose tissue were analyzed by SDS-PAGE-immunoblot assay (n = 7~8). (C) Effect of BAFF deficiency on mRNA expression related to lipid metabolism in gonadal white adipose tissue. Total RNA was isolated from inguinal and gonadal adipose tissues of mice with HF feeding and mRNA expression levels were analyzed. All genes are normalized with mRNA expression level of Arbp. Data represent means ± SD (n = 4~5). #*p <* 0.05 and ###*p <* 0.001 between male WT and male BAFF^-/-^ mice, *p < 0.05 and **p < 0.01 between female WT and female BAFF^-/-^ mice.
(TIF)

S4 Fig Female, but not male, BAFF^-/-^ mice show altered expression of thermogenic program genes in subcutaneous adipose tissue in response to cold exposure. BAFF^-/-^ and WT mice on a normal chow diet were exposed to cold (4°C) environment for 96 h. mRNA expression levels of (A) UCP1, (B) ND5, and (C) PGC1α in subcutaneous adipose tissue were measured (n = 3~5). Data represent means ± SD. *p < 0.05 between female WT and BAFF^-/-^ mice.
(TIF)
