# Supplementary figures and images for: Gender-Specific Mechanisms Underlying the Amelioration of High-Fat Diet-Induced Glucose Intolerance in B-Cell-Activating Factor Deficient Mice
Source: PLoS One. 2016 Nov 4;11(11):e0166225. doi: 10.1371/journal.pone.0166225 (PMC5096712; doi:10.1371/journal.pone.0166225)

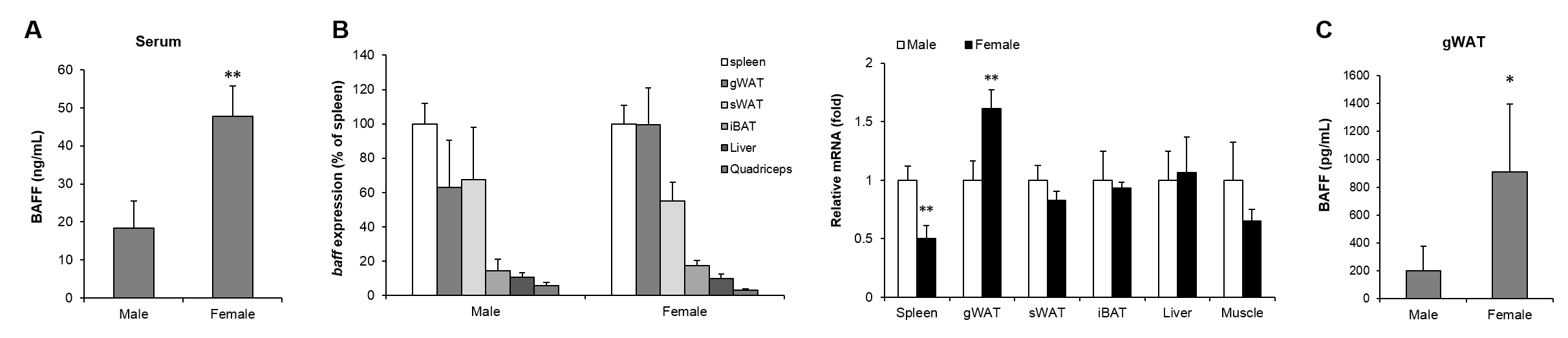

Supplement: S1 Fig — (A) Serum BAFF concentration quantified by ELISA. Serum sample were diluted 10–fold with dilution buffer, and analyzed according to the manufacturer’s protocol. (B) Levels of BAFF mRNA expression in various tissues of male and female mice. Total RNA was isolated from the spleen, liver, skeletal muscle, gonadal, inguinal, and brown adipose tissues of mice with HF feeding and BAFF mRNA expression levels were analyzed. (C) BAFF concentration in gWAT quantified by ELISA. Tissue lysates were diluted 2-fold for ELISA analysis. Total RNA was isolated from the liver, spleen, quadriceps, gonadal, inguinal, and brown adipose tissues of mice with HF feeding and BAFF mRNA expression levels were analyzed. The mRNA expression level of BAFF is normalized with mRNA expression level of Arbp. Data represent means ± SD (n = 4~5). **p < 0.01 between female WT and female BAFF-/- mice. gWAT: gonadal white adipose tissue, sWAT: subcutaneous white adipose tissue, iBAT: interscapular brown adipose tissue. (TIF) [file pone.0166225.s001.tif]

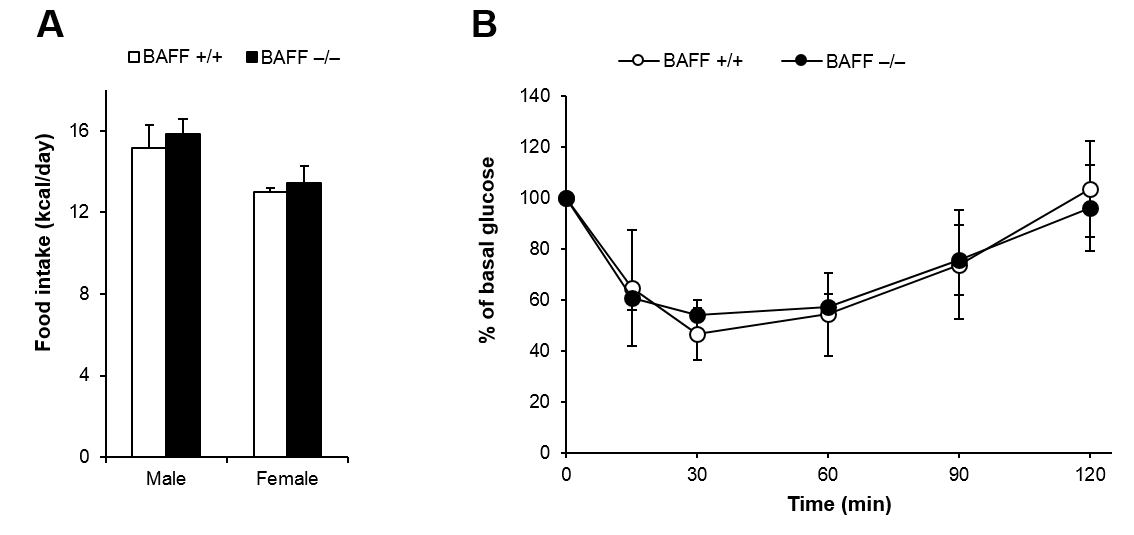

Supplement: S2 Fig — BAFF-/- and WT mice were fed a high-fat diet for 5 weeks. (A) Average daily calorie intake for 4 weeks of HFD feeding (n = 7~8). (B) Insulin tolerance of male WT and BAFF-/- mice after 4 weeks on HF diet (n = 5). The blood glucose levels were measured at 0, 15, 30, 60, 90 and 120 after intraperitoneal injection of insulin (0.75U/kg). Data represent means ± SD. (TIF) [file pone.0166225.s002.tif]

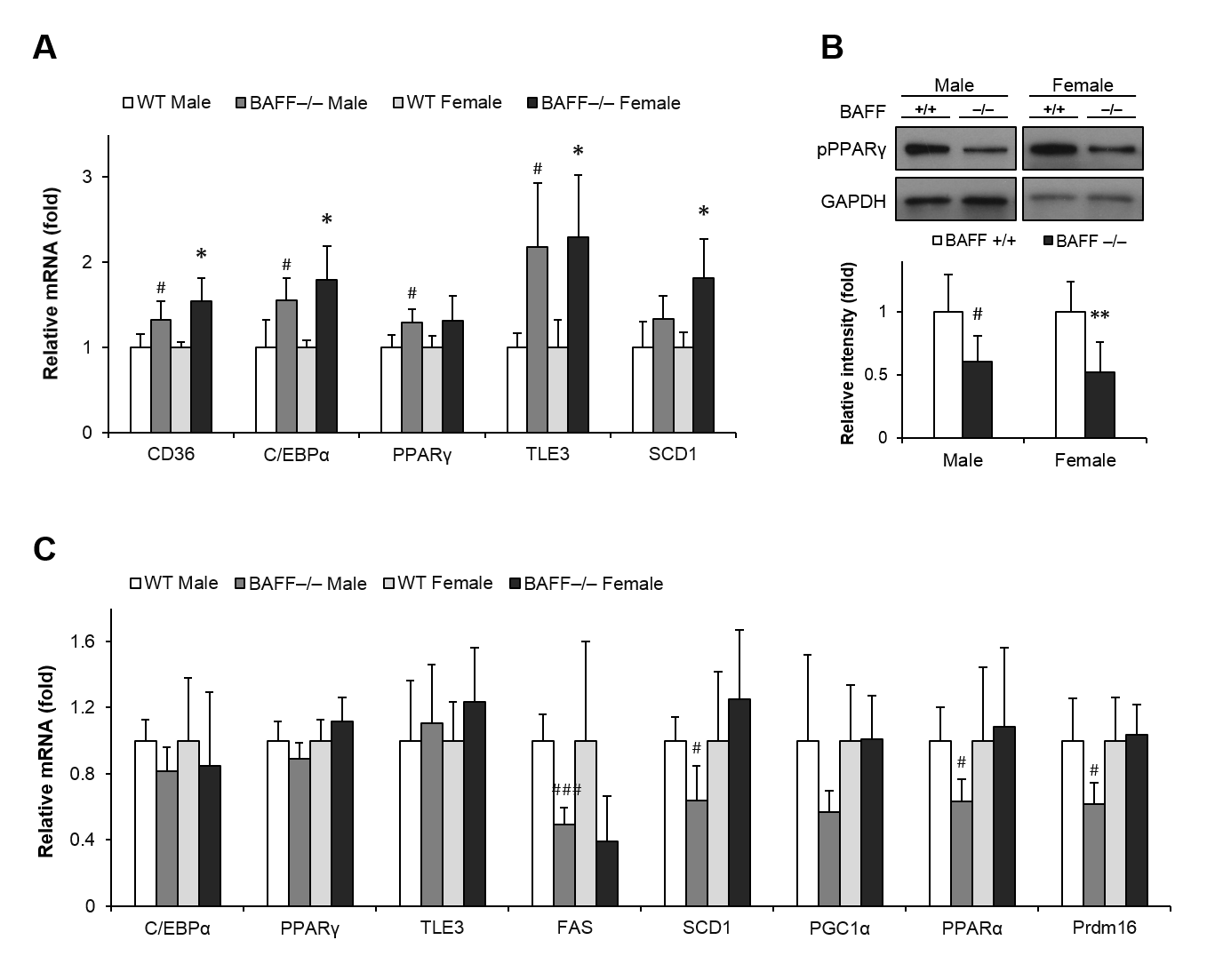

Supplement: S3 Fig — (A) Effect of BAFF deficiency on mRNA expression of lipogenic and adipogenic genes in subcutaneous white adipose tissue. (B) Effect of BAFF deficiency on PPARγ phosphorylation in subcutaneous white adipose tissue. Proteins extracted from inguinal adipose tissue were analyzed by SDS-PAGE-immunoblot assay (n = 7~8). (C) Effect of BAFF deficiency on mRNA expression related to lipid metabolism in gonadal white adipose tissue. Total RNA was isolated from inguinal and gonadal adipose tissues of mice with HF feeding and mRNA expression levels were analyzed. All genes are normalized with mRNA expression level of Arbp. Data represent means ± SD (n = 4~5). #p < 0.05 and ###p < 0.001 between male WT and male BAFF-/- mice, *p < 0.05 and **p < 0.01 between female WT and female BAFF-/- mice. (TIF) [file pone.0166225.s003.tif]

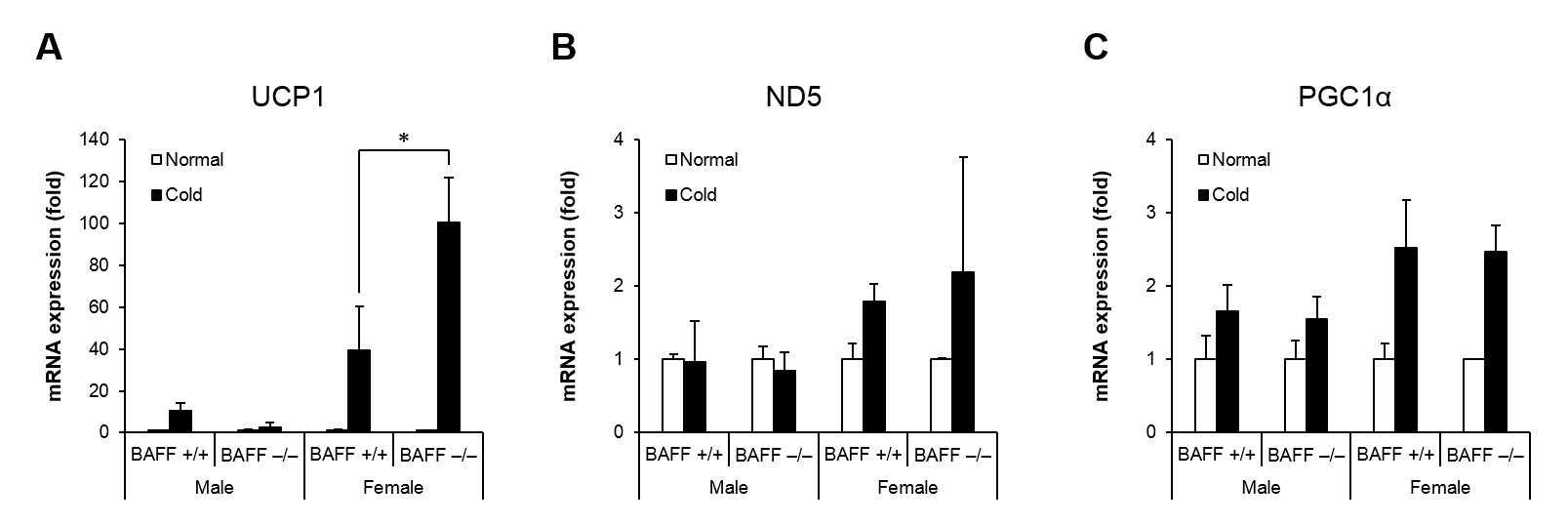

Supplement: S4 Fig — BAFF-/- and WT mice on a normal chow diet were exposed to cold (4°C) environment for 96 h. mRNA expression levels of (A) UCP1, (B) ND5, and (C) PGC1α in subcutaneous adipose tissue were measured (n = 3~5). Data represent means ± SD. *p < 0.05 between female WT and BAFF-/- mice. (TIF) [file pone.0166225.s004.tif]
